# Symptomatic irreversible pulpitis and other orofacial pain: overcoming challenges in diagnosis and management

**DOI:** 10.1038/s41415-025-8441-9

**Published:** 2025-04-11

**Authors:** David Edwards, James R. Allison, Jamie Coulter, Justin Durham, Emma V. Beecroft

**Affiliations:** School of Dental Sciences, Faculty of Medical Sciences, Newcastle University, Newcastle upon Tyne, UK; Newcastle upon Tyne Hospitals NHS Foundation Trust, Newcastle upon Tyne, United Kingdom

## Abstract

Due to the unique sensory innervation of the teeth and face, orofacial pain can be challenging to diagnose and manage. Odontogenic pain, or ‘toothache', is the most common orofacial pain condition and encompasses the vast majority of pain which is presented to dental practitioners. While diagnosis is often straightforward, the clinical picture is occasionally unclear or contradictory, and in these situations, the clinician should be able to consider reasons other than the teeth for the patient's presenting complaint. The primary aim of managing odontogenic pain is to treat the underlying cause, often arising from the dental pulp or periapical tissues; however, several factors can make pre-, intra- and post-operative management of odontogenic pain challenging. This paper will consider key similarities and differences in the clinical presentation of odontogenic pain and other non-odontogenic causes of orofacial pain in order to help practitioners arrive at the correct diagnosis. We discuss evidence-based recommendations for intra- and post-operative management of acute odontogenic pain, and consider the underlying neurophysiological features which make orofacial pain challenging to manage.

## Key points

- Orofacial pain is unique and often difficult to diagnose due to mechanisms such as peripheral and central sensitisation, presenting a diagnostic challenge.
- Other painful conditions (eg TMD) can increase the severity of, or even masquerade as, odontogenic pain.
- Effective pain management may involve systemic approaches combined with local measures to enable effective treatment and reduce the risk of post-operative pain.

## Why is orofacial pain a special case?

Orofacial pain is universally reported as severe and debilitating; symptomatic irreversible pulpitis, the most common reason for presenting for urgent dental care, is, on average, rated as 8/10 in intensity, requiring almost universal use of pain-relieving medication and time away from work.^1^^,^^2^ Given that systemic antibiotics don't work for symptomatic irreversible pulpitis, operative intervention is mandatory,^3^ bringing challenges in diagnosis and intra- and post-operative pain control.

**Fig. 1:**
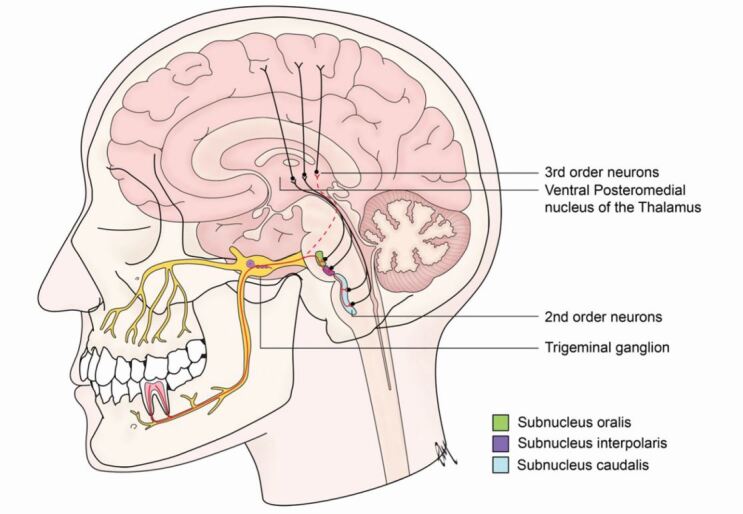
Innervation of the teeth. First-order neurons have cell bodies which reside in the trigeminal ganglion; their axons send signals from the tooth and go on to synapse with second-order neurons in the sub-nucleus caudalis of the trigeminal nuclear complex in the brainstem. Evidence from animal models suggest the presence of an additional route (dashed red line) in which primary trigeminal afferents synapse with second order neurons in the parabrachial nucleus, missing the brainstem, and offering a ‘direct' input to the brain^4^

The orofacial structures have unique sensory innervation. Most parts of the body are innervated by neurons whose cell bodies reside in the dorsal root and which synapse with second order neurons in the spinal cord. Orofacial structures, however, are innervated by the trigeminal nerve, whose cell bodies reside within the trigeminal ganglion (TG), and which synapse with second order neurons in the trigeminal nuclei in the brainstem, before ascending to synapse further in higher brain structures (thalamus, cerebral cortex). These multiple synapses allow opportunities for incoming nociceptive signals to be modified (‘turned up'/‘turned down'). An additional trigeminal pathway has also been identified, bypassing trigeminal nuclei, and feeding nociceptive signals directly into circuits involved in emotion-, fear- and instinct-related brain centres (Fig. 1).^4^ This ‘direct route' offers fewer opportunities to modify nociceptive signals and may explain why orofacial pain is often more severe and emotive than pain elsewhere in the body.^5^^,^^6^^,^^7^

## Nociception and pain

Nociception is the process by which signals about real or potential damage to tissues in the periphery (parts of the body outside of the central nervous system) are sent to the brain. The main function of nociception is to allow the brain to produce the complex phenomenon we feel as pain. Pain often produces a behaviour designed to protect the individual from harm (eg withdrawing a hand from a fire); however, pain is not always the result of harm and is not always protective.^8^ In the orofacial region, nociceptive signals may arise from an inflamed dental pulp and ultimately go on to produce the painful experience of toothache. Patients with toothache may also experience sensitivity of surrounding unaffected areas, as well as radiation of their pain to distant areas. These features are caused by peripheral and central sensitisation.

Peripherally, classical inflammation produces numerous inflammatory mediators, such as histamine, bradykinin and prostaglandins, which cause sensitisation of nociceptors (pain-sensing nerve endings) within the pulp and periodontium and produce lowered stimulation thresholds and spontaneous firing of these neurons. Additionally, activated nociceptive neurons themselves release neuropeptides which cause similar sensitisation of nociceptors in a process called neurogenic inflammation.^9^^,^^10^^,^^11^^,^^12^ Finally, activated neurons sprout more terminal branches, thus widening the field from which they receive sensation,^13^ and pulpal nociceptors which are usually dormant become activated. The net result of these peripheral mechanisms is that pain experienced is disproportionate to the stimulus (hyperalgesia) and stimuli which would not normally be painful are perceived as such (allodynia). Depolarisation of neurons without any apparent stimulus also explains the phenomenon of spontaneous pain, commonly seen in toothache. As the pulp contains few mechanoreceptors, its proprioceptive capacity is poor,^14^ and it is not until the pathological process reaches the periodontal tissues, which are well-served by mechanoreceptors,^15^ that the pain usually becomes well localised.

As well as peripheral neurons, there is evidence that central sensitisation in response to nociceptive signals from the dentoalveolar structures may also occur.^16^ At the level of the TG, activation of satellite cells, release of pro-inflammatory mediators and upregulation of N-methyl-D-aspartate (NMDA) receptors leads to further sensitisation.^17^^,^^18^^,^^19^^,^^20^^,^^21^ Given that all the cell bodies for trigeminal sensory afferents reside in the TG, it is easy to understand why, within this neuro-inflammatory environment, adjacent neurons may be affected, resulting in referred pain and further driving the reduced threshold for neuronal activation. Similar mechanisms are seen within the brainstem in the trigeminal nucleus, with the upregulation of postsynaptic NMDA receptors and activation of microglia and astrocytes, resulting in further pro-inflammatory cytokine release.^22^ These changes further add to difficulty in diagnosis due to referred pain and a widened field of perceived pain.

These central mechanisms may also drive or influence neuropathic pain, where despite the removal of a stimulus, or resolution of peripheral pathology (eg post-traumatic trigeminal neuropathic pain, discussed below) painful symptoms remain.^23^ In this scenario, while the patient may complain of toothache from a seemingly healthy tooth, nociceptive signals arising from the pulp or periodontal tissues are not driving the patient's pain, but rather a problem higher up the pain pathway.^24^

## Odontogenic pain: endodontic diagnosis

Pain of endodontic origin may currently be considered in the context of pulp and apical diagnoses, based on the American Association of Endodontists diagnostic criteria (Table 1).^25^^,^^26^ This system is simple to use and appropriate in most clinical situations, but is relatively inflexible, dividing pulpitis into dichotomous ‘reversible' or ‘irreversible' diagnoses. Any experienced dental practitioner will have managed cases which do not align perfectly with such criteria, hence the suggestion by Wolters *et al.* to introduce a more flexible system of ‘initial', ‘mild', ‘moderate' or ‘severe' pulpitis designed to align to increasing levels of intervention from simple caries management, through vital pulp treatments and finally root canal treatment.^27^

**Table 1:** Pulp and periapical diagnosis based on the American Association of Endodontists classification with associated management options^25^^,^^26^

**Diagnosis**	**Descriptor**	**Symptoms**	**Signs/further investigations**	**Management options**
**Pulp diagnosis**				
‘Normal' pulp	A clinically ‘healthy' pulp	No spontaneous pain or sensitivity reported	Under cold stimulus, pulp usually responds but pain lasts only 1-2 seconds Other sensibility testing is positive with no lingering pain	No treatment indicated
Reversible pulpitis	Inflammation of the pulp which should resolve following removal of cause (eg caries)	No spontaneous pain Discomfort from cold/sweet but pain lasts ≤2 seconds	Familiar pain following application of cold but resolution in 1-2 seconds No tenderness to percussion and no radiographic changes apically	Remove cause (eg management of caries, sealing exposed dentine). Review to ensure resolution of symptoms and maintenance of vitality
Symptomatic irreversible pulpitis	A vital inflamed pulp incapable of healing*	Sharp pain on stimulation, lasting >30 seconds (later stages may result in heat stimulus being more severe than cold) Referred pain, may be poorly located Spontaneous pain, sometimes exacerbated by postural change Pain relieving medication use and loss of sleep is frequently reported	Familiar pain lasting >30 seconds following thermal stimulation Usually positive to EPT testing (may have reduced threshold) Pain to percussion in some cases Apical radiographic changes are possible	Partial or complete pulpotomy followed by vital pulp treatment or root canal treatment Pulpectomy followed by root canal treatment Extraction
Asymptomatic irreversible pulpitis	A vital inflamed pulp incapable of healing*	No symptoms reported	Extremely deep caries and a radiograph which is likely to result in significant bacterial ingress to the pulp and therefore require pulp management	Partial or complete pulpotomy followed by vital pulp treatment or root canal treatment Pulpectomy followed by root canal treatment Extraction
Pulp necrosis	A necrotic pulp	No pulp symptoms reported but may have associated apical symptoms and diagnosis	Non-responsive to sensibility testing May have associated apical signs and diagnosis	Pulpectomy followed by root canal treatment Extraction
Previously treated	Previous endodontic treatment	No pulp symptoms reported but may have associated apical symptoms and diagnosis in the case of post-treatment disease	Non-responsive to sensibility testing May have associated apical signs and diagnosis in the case of post-treatment disease	No treatment Endodontic re-treatment Extraction
Previously initiated therapy	Endodontic treatment commenced (eg pulpotomy or pulpectomy)	Depending on the stage of treatment, symptoms may include those of symptomatic irreversible pulpitis or pulp necrosis	Depending on the stage of treatment, signs may include those of symptomatic irreversible pulpitis or pulp necrosis	Root canal treatment Extraction
**Apical diagnosis**				
‘Normal' apical tissues	No inflammatory or infective process	Any symptoms present are likely to be pulpal origin only	No sensitivity to percussion Normal lamina dura radiographically with normal PDL space	No treatment If pulpal symptoms, management aligning to this
Symptomatic apical periodontitis	Periapical inflammation secondary to pulpitis or infection of the root canal system	Patients may report pain on biting of short duration Pain is well-located Spontaneous pain is possible	Tooth usually tender to percussion Periapical radiolucency or hypoattenuation may be seen on radiographs/CBCT scans	Vital pulp treatment Extirpation and root canal treatment Endodontic re-treatment Extraction
Asymptomatic apical periodontitis	Periapical inflammation secondary to pulpitis or infection of the root canal system	No additional symptoms to pulp diagnosis May be asymptomatic (eg previously treated, pulp necrosis)	Tooth not tender to percussion Periapical radiolucency or hypoattenuation may be seen on radiographs/CBCT scans	Vital pulp treatment Extirpation and root canal treatment Endodontic re-treatment Extraction
Chronic apical abscess	Usually gradual onset, pus is produced in response to intra and/or extra radicular infection. Sinus is present	Usually asymptomatic Patients may be aware of sinus/bad taste	Non vital/previously treated tooth Presence of a sinus adjacent to tooth Tooth mobility possible Periapical radiolucency or hypoattenuation may be seen on radiographs/CBCT scans which is well defined and may be corticated Root resorption is possible in long standing cases	Extirpation and root canal treatment Endodontic re-treatment Extraction
Acute apical abscess	Pus is produced in response to intra and/or extra radicular infection. May be exacerbation of chronic condition (eg chronic abscess, granuloma, cyst) or acute aetiology	No pain to thermal stimuli Usually well-located pain which is spontaneous and is exacerbated by pressure	Non-vital/previously treated tooth Tooth mobility is possible Swelling May be pus discharge from socket/sinus	Extirpation and root canal treatment Endodontic re-treatment Extraction Antibiotics if drainage not possible and/or systemic involvement
Condensing osteitis	A localised bony reaction to low-grade long-standing inflammation	May be present secondary to other diagnosis, with symptoms aligning to this When present in isolation, often asymptomatic	May be present secondary to other diagnosis, with signs aligning to this Periapical radiograph shows radiopacity around root apices	Extirpation and root canal treatment Endodontic re-treatment Extraction.
Key: * = Irreversibly inflamed pulp tissue may be confined to part of the pulp, the remainder being amenable to vital pulp treatments EPT = electric pulp test PDL = periodontal ligament CBCT = cone-beam computed tomography				

Whichever diagnostic system is favoured, extensive clinical evaluation is needed. This starts with a detailed pain history, followed by clinical examination aimed at identifying non-odontogenic causes of pain (eg TMD), the presence of swelling or a sinus, occlusal factors, periodontal diagnoses (eg narrow pocket suggesting root fracture, endodontic-periodontal lesions), pulp diagnosis (using sensibility tests or vitality tests) and apical diagnosis (through percussion testing and further investigations such as periapical radiography or cone-beam computed tomography [CBCT]). To test for pulp vitality, surrogate measures such as thermal testing and electronic pulp testing (EPT) have excellent sensitivity and specificity, especially when combined.^28^ In contrast, percussion testing has a lower sensitivity and specificity. True measures of pulp vitality, such as measures of blood flow (eg pulse oximetry), have up to 98% sensitivity and specificity but are not yet widely available.^29^^,^^30^

Unlike pulp diagnosis, the apical diagnosis is often more straightforward, with more localised pain and other signs such as a lack of response to sensibility tests, tenderness to percussion, and signs of bone destruction on a radiograph or CBCT.

## Intra-operative management of odontogenic pain

Management of intra-operative pain to facilitate operative acute endodontic care is a priority for clinicians and patients. Positive intra-operative management has also been shown reduce the risk of persistent pain following endodontic treatment and so is critical for immediate management and longer-term outcomes.^31^ However, achieving anaesthesia is often more challenging for patients with acute dental pain.^32^^,^^33^ Peripherally increased levels of inflammatory mediators, phenotypic changes in ion channels, nociceptor activation and sensitisation, and nerve sprouting all account for increased neuronal activity even in the presence of local anaesthetic (LA) solutions. Furthermore, peripherally applied LA solutions would not be expected to attenuate central sensitisation which will continue to exaggerate nociceptive responses to peripheral stimuli.^34^ For those with swelling, distortion of anatomical landmarks, trismus and tissue acidity further complicate the process of anaesthesia. Overcoming anaesthetic difficulties can be considered in terms of anaesthetic agents, supplementary techniques and systemic approaches (Box 1).

Box 1 A summary of key clinical points around intra-operative pain management

- Positive intra-operative pain management supports short- and long-term pain control
- Consider, where appropriate, a single dose of ibuprofen 60 minutes pre-treatment for those with severe/prolonged pain or at high risk of persistent pain
- Carefully select local anaesthetic techniques to optimise pain management
- Where pulpectomy cannot be predictably achieved, a pulpotomy offers effective pain relief
- Patient-clinician interactions and the clinical environment have an effect on pain control.

## Anaesthetic agent

In addition to simply increasing volume,^35^ administering 4% articaine with 1:100,000 adrenaline for inferior alveolar nerve block (IANB) and buccal infiltration has been shown to be more effective than using 2% lidocaine with 1:80,000 adrenaline,^36^^,^^37^ especially in cases of symptomatic irreversible pulpitis.^38^ The use of 4% articaine for inferior alveolar nerve block (IDB) has been a contentious issue over the last couple of decades, due to reported increased incidence of sensory disturbance compared to 2% lidocaine.^39^^,^^40^ However, recent high-quality evidence suggests there may be no increased risk of sensory disturbance using 4% articaine compared to 2% lidocaine, therefore given its therapeutic benefit, this approach is now worthy of consideration.^41^ Additionally, supplementary techniques such as intraosseous injections have been shown in large studies to have superior anaesthetic success rates to alternative techniques, and may therefore be useful in cases of difficult-to-achieve anaesthesia.^42^

## Supplementary techniques

A range of supplementary techniques, outlined in Table 2, can be used to support anaesthetic success. These include supplemental infiltrations with 4% articaine,^43^ intraligamentary, intraosseous, or intra-pulpal injections, and high mandibular blocks.^34^

**Table 2:** Approaches for effective dental anaesthesia

**Approach**	**Upper arch**	**Lower arch**
First line	4% articaine with 1:100,000 adrenaline buccal and palatal infiltration total volume 3.2 mL	Conventional IDB and lingual nerve block with 2% lidocaine with 1:80,000 adrenaline, total volume 2.2 mL and 4% articaine with 1:100,000 adrenaline buccal infiltration, total volume 2.2 mL
Second line	4% articaine with 1:100,000 adrenaline buccal and palatal infiltration, total volume 3.2 mL	Repeat conventional IDB and lingual nerve block with 2% lidocaine with 1:80,000 adrenaline 2.2mL or consider IDB and lingual nerve block with 4% articaine with 1:100,000 adrenaline, total volume 2.2 mL
	2% lidocaine with 1:80,000 adrenaline as intra-ligamentary injection up to 1.4 mL	4% articaine with 1:100,000 adrenaline lingual infiltration, total volume 2.2 mL
Further options	Intra-osseus infiltrations using 2% lidocaine with 1:80,000 adrenaline, up to 1.4 mL	2% lidocaine with 1:80,000 adrenaline as intraligamentary injection, up to 1.4 mL
	Regional block techniques using up to 2.2 mL 2% lidocaine with 1:80,000 adrenaline can be helpful in the presence of swelling in the region of normal infiltration position Infraorbital nerve blocks Greater palatine nerve block Nasopalatine nerve block	High block techniques (Vazirani-Akinosi/Gow-Gates technique) 2% lidocaine with 1:80,000 adrenaline, total volume 2.2 mL
		Mental nerve block using up to 2.2 mL 2% lidocaine with 1:80,000 adrenaline can be helpful in the presence of swelling in the region of normal infiltration position
	Intra-pulpal infiltration using any local anaesthetic up to 0.5 mL	
Long-acting	Levobupivicaine 2.5 mg/mL (0.25%) or 5 mg/mL (0.5%) solution as IDB, or infiltrations up to 5 mL Provided upon completion of operative procedure, where appropriate, for enhanced duration of post-operative pain control	

## Systemic approaches

Some studies have highlighted the use of pre-operative systemic non-steroidal anti-inflammatory drugs (NSAIDS) or corticosteroids (individually or in combination) as being beneficial for intra-operative anaesthesia and post-operative pain control.^44^ In particular, evidence from systematic reviews suggests that NSAIDs such as ibuprofen taken pre-emptively before a procedure improve the success rate of LA in cases of symptomatic irreversible pulpitis,^45^^,^^46^ and have the additional benefit of reducing prostaglandin synthesis, thereby reducing the potential for post-operative inflammation and sensitisation. Although ibuprofen is the most widely used NSAID in dentistry in the UK, other NSAIDs have been associated with a greater effect, including indomethacin, meloxicam, diclofenac potassium and piroxicam, but may not have gained in popularity due to different adverse effect profiles.^45^ Further, at this present time, only ibuprofen and diclofenac sodium are listed on the dental practitioner's formulary; therefore, in clinical practice, individuals attending in pain could be advised, when safe to do so, to take 600 mg oral ibuprofen or 50 mg diclofenac sodium one hour before their appointment time to maximise efficacy of LA treatment.^47^ This could be particularly beneficial for individuals at increased risk of the development of persistent pain, to pre-emptively mitigate inflammatory processes. Evidence review could lead to the addition of further NSAIDs to the dental practitioner's formulary providing the opportunity of a wider breath of pain management options for dental clinicians in the future.

## Pulpal treatment

Once effective anaesthesia is achieved, treatment options for symptomatic irreversible pulpitis may include partial or complete pulpotomy, or pulpectomy. Where resources allow, it is desirable to aim for a ‘definitive' pulpotomy using calcium silicate cements. This is discussed in detail in the European Society of Endodontology position statement on deep caries and the exposed pulp.^45^ It is worth noting that ‘definitive' pulpotomies have been shown to offer effective pain relief within a few days, emphasising their suitability for first line treatment in suitable cases.^48^

In cases where a vital pulp treatment (eg pulpotomy using calcium silicate cement) is not feasible, due to resource limitations or uncontrollable pulpal haemorrhage, it may be desirable to undertake a pulpectomy. However, Eren (2018) found a pulpotomy is as effective as a pulpectomy for the management of pain in symptomatic irreversible pulpitis.^49^ It is therefore advisable in the resource-limited urgent dental appointment, and/or where pulpectomy is challenging, that a pulpotomy is undertaken. Further uncertainty exists around the use of medicaments to manage pain. Two UK-based studies found that around two-thirds of practitioners currently place antibiotic/corticosteroid dressings into teeth following pulpotomy or pulpectomy.^50^^,^^51^ For pain relief in symptomatic irreversible pulpitis, there is no clear benefit of using such dressings above simple excision of irreversibly inflamed tissue and the placement of damp cotton wool or calcium hydroxide,^52^ suggesting the latter is appropriate, especially in the current climate of antimicrobial stewardship.^53^

In cases of acute or chronic abscess, although achieving anaesthesia can be challenging, drainage of infection through incision and/or extirpation and instrumentation to gain apical patency is usually effective.

## Clinical experience

Our ability as clinicians to manage the patient experience can have a significant effect on pain management. Nixdorf *et al.* showed that patient treatment expectation being ‘very good' resulted in 61% decreased risk of persistent pain following root canal treatment.^54^ Environmental factors that clinician's control can also support positive pain management. For example, creating a relaxing environment with instrumental music has been shown to reduce intra- and post-operative pain and anxiety during surgical dental treatment.^55^

## Post-operative pain management

Despite best efforts, a recent study undertaken in primary care suggests that one in four patients managed for symptomatic irreversible pulpitis may return due to pain within seven days.^52^ It is likely that the combined effect of peripheral and central sensitisation drives this finding. Positive post-operative pain management is important for every patient, but particularly so for those at increased risk of persistent pain conditions (Box 2).

Box 2 A summary of key clinical points to optimise post-operative pain management

- Discussing and planning for post-operative discomfort should form an important part of the consent process and clinical treatment
- Individually determined, high-quality analgesic advice should be provided following endodontic treatment
- Careful chemo-mechanical disinfection followed by definitive restoration will maximise endodontic success while minimising post-operative pain
- Concomitant pain should be discussed, and supportive management advice provided as part of holistic post-operative pain management plan.

## Oral analgesics

Analgesic advice for patients with odontogenic pain should revolve mainly around NSAIDs such as ibuprofen. Given the role of prostaglandins in pulp inflammation and neuronal sensitisation outlined earlier, this class of drugs are the most effective option.^56^ Combining ibuprofen with paracetamol appears to offer enhanced analgesia over NSAIDs alone, with the effect being synergistic rather than merely additive,^45^^,^^57^ although a recent systematic review found this effect was not significant unless combined with caffeine.^56^ Other NSAID drugs have been studied, including Naproxen and Ketoprofen, which both reduced post-operative endodontic pain more than ibuprofen, although this was not significant.^56^ This perhaps highlights the need for further research in this area.

In contrast, centrally acting analgesics such as opioids (eg codeine) offer limited benefit in odontogenic pain, even when combined with NSAIDs.^58^ Given their adverse effect profile, they are best avoided. Optimising post-operative dosage (where appropriate, short courses of 600 mg oral ibuprofen TDS or QDS in preference of 400 mg doses) and improved patient advice (leaflets or patient-specific letters outlining analgesic advice) have been shown to significantly improve post-operative pain levels.^59^

In addition to the benefits of pre-operative administration of NSAIDs in enhancing LA effectiveness discussed earlier, there are further benefits of pre-operative administration for post-operative pain experience. Their use pre-operatively has been shown to be an important predictor for reduced post-operative pain following the endodontic management of symptomatic irreversible pulpitis.^45^ One under-used approach may be to administer oral corticosteroids pre-operatively, which has been shown to reduce post-operative pain scores by up to 30%.^60^

## Operative procedures

Root canal treatment in the absence of infection, such as cases of symptomatic irreversible pulpitis, may be best managed in a single visit.^61^^,^^62^ Careful technique, such as minimising the extrusion of potentially infected dentine debris and thorough chemo-mechanical disinfection, may minimise the risk of flare-up, post-operative pain and post-treatment disease.^63^^,^^64^ The provision of a definitive restoration as soon as possible may also minimise the risk of reinfection and enhance tooth survival in the long run.^65^ Upon completion of an operative procedure, application of a long-acting LA (eg levobupivacaine) can be considered to enhance duration of post-operative pain control (Table 2).^66^

## Non-odontogenic orofacial pain

While most commonly odontogenic in origin, orofacial pain may also be caused by a musculoskeletal, neuropathic (nerve-based), neurovascular (eg migraine) or idiopathic process. Clinicians should hold a high index of suspicion for signs and symptoms suggestive of pain of non-odontogenic origin, which can present either at the patient's primary or concurrent pain source (Box 3).

Box 3 Key clinical points for TMD management^71^

- Rule out TMD as primary cause or contributing factor in suspected odontogenic pain
- The most common non-odontogenic cause of pain following root canal treatment is TMD
- Manage the temporomandibular joint apparatus with care when completing endodontic treatment, especially in those with pre-existing TMD:
  - Gentle manipulation
  - Rest breaks or space procedure out over a few visits
  - Bite blocks (‘mouth prop') sized to less than maximal opening
  - Pre-operative analgesics
  - Post-operative TMD supported self-management advice (rest, heat or covered ice application, facial massage, modified diet).

## Temporomandibular disorders

Considering their prevalence, it is probably unsurprising that temporomandibular disorders (TMD) are frequently misdiagnosed as odontogenic pain. A recent study investigating patients diagnosed with odontogenic pain in an endodontic department found that 8% of cases had TMD as the sole aetiology (no odontogenic cause), and 20% had TMD as a major factor in a combined aetiology.^67^ TMD pain symptoms (dull, throbbing ache) may mimic odontogenic pain and pain in TMD may refer to molar (mandibular>maxillary) and premolar (maxillary>mandibular) teeth, leading to diagnostic difficulty.^68^ Endodontic treatment itself often exacerbates TMD symptoms due to prolonged mouth opening, and following endodontic treatment, prolonged pain form an odontogenic cause (treated or adjacent tooth) is equally prevalent to pain from TMD.^69^ Such findings highlight the importance of clinicians considering the following points:

- Could TMD be the primary causative factor of the patient's complaint?
- Could an individual's TMD be contributing to their current pain experience?

Fortunately, identification of TMD is relatively straightforward. Box 4 provides an examination summary to help clinicians identify TMD. For detailed diagnostic and management advice, referral to the recently published guideline *Management of painful TMD in adults* is advised.^70^

Box 4 Clinical examination to identify temporomandibular disorder^70^^,^^88^ **Identification of arthrogenous TMD:**

- Use two fingers to apply direct pressure over the lateral pole of the temporomandibular joint through three cycles of opening and closing and lateral excursive movements
- Positive findings: identification of pain and/or functional joint issues such as joint noises or restriction of mandibular motion.

**Identification of myogenous TMD:**

- Palpation of the origin, body, and insertion of temporalis and masseter (bimanually where possible)
- Positive findings: identification of familiar pain when palpating masseter and or temporalis.

**Key examination concepts:**

- Familiar pain: positive confirmation of pain which is representative of the patient's actual complaint. If pain is present, but it does not correspond to the complaint, this most likely represents TMD presence which is secondary to the primary pain issue
- Pain modification: pain modification with jaw use, ie worsening of pain with jaw movement or improvement of pain with jaw rest, would be an expected feature of TMD, but would not be expected for pain of either odontogenic or neuropathic origin.

## Post-traumatic trigeminal neuropathic pain

Persistent pain following endodontic treatment is a risk which can present regardless of the quality of the endodontic therapy. Post-traumatic trigeminal neuropathic pain (PTTN) is a rare condition following a preceding injury to trigeminal nerve fibres, which may include endodontic treatment, and can present in relation to a tooth with clinical and radiographic evidence of an otherwise satisfactory outcome (Box 5). Table 3 outlines key features and prevalence of PTTN. Early identification is important to avoid unnecessary interventive treatment, with an examination checklist suggested in Appendix 1. Though screening instruments show some potential, at present, there is no definitive diagnostic test. Combining symptomatic features and clinical examination findings can help to increase or decrease diagnostic suspicion.^72^ Any suspected case should be referred to a specialist care setting at the earliest point to optimise outcome.^73^

**Table 3:** Summarised features of PTTN^24^^,^^69^^,^^89^^,^^90^^,^^91^^,^^92^

**Historical terminology: anaesthesia dolorosa, painful post-traumatic trigeminal neuropathy**
**Simplified definition**
Pain within the distribution of trigeminal nerve(s), persisting or recurring for >3 months Identifiable causative event
**Epidemiology**
Women > men Late-middle-aged (40-50 years old) High psychosocial burden and negative quality of life impact
**Presentation**
Difficult to describe Can mimic odontogenic pain Sleep unaffected Mild to moderate pain intensity
Well-localised Most common descriptors: burning, itching Usually continuous Rarely presents as paroxysmal pain which can be spontaneous or triggered by function or touch
**Incidence**
0.3-1.6% following root canal treatment 5% following apicectomy 8% following dental implant placement 3.3% following major facial trauma
**Somatosensory change**
Will always have somatosensory symptoms and/or signs in the same distribution of trigeminal nerve as pain is presenting

Percussion tests are not usually helpful in differentiating odontogenic pain and PTTN; however, tenderness to apical palpation suggests the presence of inflammatory dental disease (eg apical periodontitis).^74^^,^^75^ The presence of somatosensory change(s) are particularly helpful, including alterations in patients' perception of touch, pain, pressure and temperature. Both gain in sensation (eg hyperalgesia or allodynia) or reduction in sensation (eg hypoalgesia or paraesthesia) is observed compared to the unaffected side, and a difference is considered abnormal. Somatosensory abnormalities suggest a non-odontogenic cause such as PTTN and are rare in odontogenic pain or the absence of pain.^76^^,^^77^^,^^78^^,^^79^ Qualitative sensory testing (Box 6) allows chairside assessment of somatosensory change and does not require complex equipment.^80^

Box 5 Key diagnostic features of PTTN

- Unusual pain descriptors
- Pain persistence beyond normal timeframe of healing
- Repeated ‘failed' dental interventions
- Sleep unaffected
- Evidence of somatosensory change with chairside qualitative sensory testing.

Box 6 Chairside qualitative sensory testing Touch:

1. Hold cotton wool roll gently against the gingivae adjacent to the affected/painful site of the dentoalveolus for three seconds
2. Repeat the procedure on a contralateral, equivalent, pain-free (control) site
3. Ask the patient whether at the painful site the sensation was more (gain), less (loss), or the same as the control site.

Pain and temperature:

Repeat stage 1, 2 and 3 above using:

- Something slightly sharp such as a periodontal probe
- Something cold (eg cotton wool with coolant applied or cooled mirror handle).

## Risk factors for development of persistent orofacial pain

An awareness of population groups most at risk of persistent orofacial pain can help support diagnosis, inform the consent process and mitigate risk of development of persistent pain. The following features are associated with an increased risk of persistent orofacial pain (Box 7).

Box 7 Risk factors and suggested management of persistent orofacial pain

- Increased pre-operative pain intensity and pain duration, repeated dental treatments, female sex, current or historic comorbid pain conditions and psychosocial comorbidity are all risk factors for the development of persistent orofacial pain
- Consider and discuss individual risk factors when consenting patients for endodontic treatment
- Comprehensive treatment planning should avoid the need for repeated procedures
- Swift, definitive endodontic management for any individual with odontogenic pain supports improved short- and long-term pain control
- Where possible, stabilise comorbid conditions eg TMD before endodontic treatment.

### Duration and intensity of pain

Increased duration and intensity of odontogenic pain before endodontic treatment can adversely affect long-term outcome, with an increased risk of PTTN development.^31^^,^^54^ Nixdorf *et al.* demonstrated that for each additional day of pain experienced before endodontic treatment, there was a 19% increased risk of persistent pain.^54^ Swift, definitive management of odontogenic pain would therefore be expected to improve both short- and long-term patient outcomes.

### Sex

Clinical studies demonstrate a four-fold increased risk of PTTN for women compared to men, while women have been shown to be marginally more affected by TMD than men.^31^^,^^74^^,^^81^ This phenomenon is not isolated to the orofacial region; persistent pain is more common in women and sex differences in pain reporting, perception, and endogenous pain modulation are accepted, though not yet fully understood.^82^

### Comorbid pain

The experience of patients reporting worsening concomitant trigeminal pain issues, eg ‘my toothache has been so bad it has triggered my migraine', will have been experienced by many clinicians. In the context of comorbid pain, where induced changes in an individual's nociceptive system as a result of peripheral and central sensitization are pre-existing, the additive nociceptive burden effectively amplifies nociceptive messages, exacerbating the experience of each painful condition. One of the most common comorbidities which can exacerbate endodontic pain is TMD, as discussed previously.^67^ Following root canal treatment, those with a comorbid pain condition are 4.5 times more likely to develop persistent pain (PTTN).^31^

Other subtle features in an individual's history such as the presence of systemic or regional pain conditions (eg fibromyalgia), or a history of persistent pain/reports of ‘nerve damage' following surgery can suggest more generalised issues in an individual's pain processing, increasing the risk of persistent pain following treatment.

### Psychosocial comorbidities

Depression and anxiety directly result in dysregulation of chemical messengers and neurotransmitters implicated in trigeminal pain.^83^^,^^84^^,^^85^^,^^86^ Indirectly, such conditions may be associated with reduction in self-care practices, healthcare avoidance, poor diet, and parafunctional habits. The resultant consequence is that those with psychosocial comorbidities such as stress, anxiety, depression, fear, or avoidance behaviours have been shown to display higher levels of pain intensity and pain related disability with respect to odontogenic and non-odontogenic pain conditions.^87^

## Conclusion

While in many cases, managing odontogenic pain is straightforward, even the experienced clinician can be caught out by an unusual presentation. It is important that dental practitioners take time to consider alternative, non-odontogenic causes of pain where clinical features do not accord with a diagnosis of ‘toothache'. While management of an irreversibly inflamed pulp or apical abscess may be the primary goal of treatment, and the most important factor to alleviate the patient's pain, close attention to effective and individualised intra- and post-operative pain management may vastly improve the patient's experience of treatment and their clinical outcomes in the short- and long-term.

Appendix 1 Assessment for suspected PTTN in primary care setting **History**

Onset, duration, frequency, pattern, characteristics, intensity, quality, potential causative event in last 6/12M. PTTN most commonly burning/shooting pain, well localised.

**Associated features**

- Stress exacerbations
- Effect on sleep.

**Medical and social history**

- Full systems check
- Comorbidities
- Medications
- Allergies
- Social history.

**Cranial nerve exam**

At a minimum, trigeminal and facial nerve exam to rule out other pathology. Any positive finding should result in urgent referral to appropriate secondary care specialist team for assessment.

**Rule out red flags**

Any positive red flag finding should result in urgent referral to appropriate secondary care specialist team for assessment:

- History of previous malignant tumour with facial pain or headache
- Lymphadenopathy, face or neck mass/swelling
- Systemic symptoms (weight loss, fever)
- Pyrexia, swelling, trismus
- Neurological signs/symptoms:
  - Acute onset loss of smell or hearing
  - Acute onset visual problems
  - Neurosensory change
  - Motor function changes.
- Pain with exertion, coughing or sneezing. (suggests raised intracranial pressure)
- Nasal symptoms (persistent and profuse bleeding or [purulent] discharge)
- Hoarse voice/voice change
- Dysphagia
- S/T lesion (E/O or I/O)
- New onset jaw pain in those taking bisphosphonates or related medication.

**E/O and I/O examination**

1. Lymph nodes
2. Neck Face
3. Muscles of mastication
4. Temporomandibular joint
5. Major salivary glands
6. Intra oral soft tissue screening.

**Dental assessment**

- Detailed clinical assessment
- Pulp vitality testing
- Tooth sleuth to assess for potential cracked cusp(s)
- Qual ST (somatosensory assessment).

**Imaging**

- Plain film radiographs:

As needed to rule out dental pathology.

- CBCT:
  - In area of pain to rule out dental or bony pathology not visible on plain film images or with clinical examination
  - Following root canal treatment can assess quality of obturation and presence of supplemental canals which could prove potential odontogenic cause of pain.
